# Spinach yield and quality response to elevated soil carbon dioxide

**DOI:** 10.3389/fpls.2025.1636651

**Published:** 2025-11-06

**Authors:** Ying Huang, Xueyan Zhang, Xin Ma

**Affiliations:** 1 Key Laboratory of Land Surface Pattern and Simulation, Institute of Geographic Sciences and Natural Resources Research, Chinese Academy of Sciences, Beijing, China; 2 University of Chinese Academy of Science, Beijing, China; 3 Institute of Environment and Sustainable Development in Agriculture, Chinese Academy of Agricultural Sciences, Beijing, China

**Keywords:** spinach, simulated experiment, quality change, CO_2_ leakage, carbon capture and storage

## Abstract

**Introduction:**

With the widespread implementation of carbon capture and storage (CCS) projects, assessing the associated environmental risks has become increasingly important, particularly concerning crop responses to soil carbon dioxide (CO_2_) leakage. While previous studies have examined plant responses to soil CO_2_ stress, the implications for crop nutritional quality remain poorly characterized.

**Methods:**

A pot experiment was conducted in Shunyi, Beijing, during autumn 2023 to quantify the effects of CO_2_ leakage from CCS on the nutritional indicators of leafy vegetables. The experiment included four replicates per treatment, consisting of a control group (CK) and a CO_2_ leakage treatment group (1500 g·m^-2^·d^-1^, G1500). Spinach yield and quality were assessed under elevated soil CO2 conditions, with emphasis on vitamin C, vitamin E, cellulose, and oxalate content.

**Results:**

Prolonged exposure to high soil CO2 concentrations severely inhibited spinach growth, reducing leaf area, aboveground fresh weight, and root weight by 92.76%, 93.46%, and 95.83%, respectively. Chlorophyll b decreased by 35.48%, indicating impaired photosynthesis. Conversely, concentrations of vitamin C, vitamin E, and cellulose increased by 185.47%, 131.45%, and 315.03%, respectively, while oxalate content decreased by 43.08%. However, the severe biomass reduction led to an overall decline in total nutrient yield per plant.

**Discussion:**

These findings demonstrate that soil CO_2_ leakage markedly inhibits growth and reduces total nutrient yield in leafy crops, despite the relative enrichment of certain nutritional components. The results highlight critical challenges to agricultural productivity and food quality in regions affected by CO_2_ leakage from CCS.

## Graphical Abstract

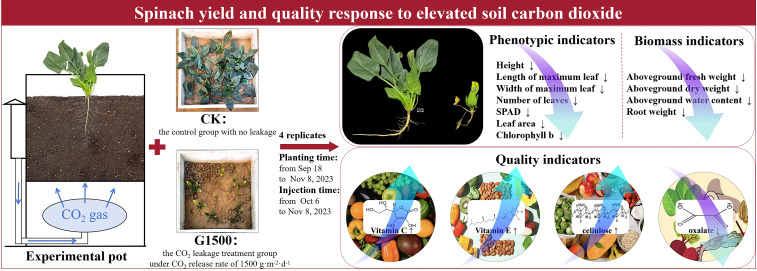

## 1 Introduction

Carbon capture and storage (CCS) technology effectively reduces carbon dioxide (CO_2_) emissions from primary pollution sources and is one of the key technologies to achieve global net-zero emission goals (IPCC, 2023; Chen et al., 2024). CCS involves capturing, transporting, and storing CO_2_ in geological formations (Bukar and Asif, 2024). However, CO_2_ may leak through wells or faults, as stable mineralization rarely occurs (Wu et al., 2024; Xiao et al., 2024), resulting in a persistent risk of leakage (Liu et al., 2022). Thus, assessing leakage risks is essential before large-scale CCS deployment (Zhang et al., 2022). This study focused on the impact of high soil CO_2_ concentrations from CCS leakage on plant growth. CO_2_ leakage can displace soil oxygen, inhibiting root respiration and reducing biomass (Maček et al., 2005). Natural leaks at Mammoth Mountain caused tree mortality (Farrar et al., 1995), and Mediterranean pastures near vents showed no plant growth within 6 meters, although recovery occurred farther away (Beaubien et al., 2008). As sedimentary basins suitable for CCS frequently overlap farmland, the potential impact of elevated soil CO_2_ concentrations on farmland has garnered considerable attention. Projects such as ASGARD (Artificial Soil Gassing and Response Detection), Ginninderra, and ZERT (Zero Emission Research and Technology) simulated leaks to study the responses of crops. ASGARD found that CO_2_ injection at a depth of 60 cm inhibited field bean growth and increased mortality due to hypoxia (Al-Traboulsi et al., 2012). Similarly, Zhang et al. (2024) observed reduced chlorophyll levels in wheat under CO_2_ leakage conditions, which correlated with the leakage rate. Lake and Lomax (2018) reported that impurities like SO_2_ and H_2_S in leaks had no significant additional impact compared to pure CO_2_. Crops such as maize, clover, and alfalfa show reduced biomass beyond certain CO_2_ thresholds (Zhang et al., 2018). However, effects on quality vary: alfalfa showed declines in crude protein and amino acids (Zhang et al., 2022), whereas sunflowers increased in crude protein and linoleic acid under leakage (Yu et al., 2020). Further research is required to assess the effects of CCS CO_2_ leakage on crop quality and explore the underlying mechanisms. Plants respond to external stresses through intricate physiological, molecular, and biochemical mechanisms (Bashir et al., 2021), mitigating adverse effects on growth, development, and reproduction (Hasanuzzaman et al., 2013; Ma et al., 2020a). These stress conditions often lead to the accumulation of free radicals and reactive oxygen species (ROS) in plant cells (You and Chan, 2015). To counteract this, synthesizing antioxidants such as vitamins C and E plays a crucial role in scavenging excess ROS, thereby protecting cells from oxidative damage (MacGregor, 2024). Moreover, flavonoids, a class of secondary metabolites, exhibit significant antioxidant properties, further aiding plants in counteracting ROS-induced damage (Macedo et al., 2024). In addition to these biochemical defenses, the cell wall serves a critical function under stress by enhancing its mechanical strength to maintain cellular rigidity and stability (Swaminathan et al., 2022; Zhao et al., 2024). Furthermore, environmental stress can alter the oxalate levels in plants, which may, in turn, be linked to changes in carbon metabolism (Zhou et al., 2019) and antioxidant mechanisms (Shi et al., 2024). Therefore, investigating the effects of soil CO_2_ stress on the contents of vitamins, flavonoids, cell walls, and oxalate provides valuable insights into the mechanisms underlying plant responses to CO_2_ leakage from CCS.

Spinach (*Spinacia oleracea L.*), known for its high sensitivity to environmental stress, is an ideal vegetable for studying stress tolerance and adaptation mechanisms (Badar et al., 2024). Rich in vitamins, carotenoids, folic acid, and minerals, spinach is an important leafy vegetable with high nutritional value (Watanabe and Ayugase, 2015) and is widely consumed globally (Xu et al., 2017). However, spinach is prone to accumulating nitrates and oxalates during growth, and excessive consumption of spinach containing these substances may pose potential health risks (Ghanati et al., 2024; Khaksar et al., 2024). The effects of elevated atmospheric CO_2_ on plant growth have been extensively studied (Genthon et al., 1987; Liu et al., 2018). In studies on spinach under high CO_2_ conditions, researchers have primarily focused on FACE (Free-Air CO_2_ Enrichment) experiments, investigating the impact of elevated atmospheric CO_2_ (eCO_2_) on spinach morphology, biomass, and quality. Ahmad et al. (2024) found that under eCO_2_ conditions, spinach root length and stem length increased significantly by 36.76% and 20.42%, respectively, and the fresh weight, dry weight, leaf number, and leaf area all showed growth. Increased atmospheric CO_2_ promoted photosynthetic activity, enhancing crop productivity (Ma et al., 2021). In a meta-analysis of leafy vegetables, Dong et al. (2020) also found that spinach yield increased by 35% under eCO_2_ conditions. Giri et al. (2016) evaluated the effects of eCO_2_ concentrations on spinach growth and nutritional quality, revealing a significant decrease in leaf stomatal conductance and a reduction in the concentrations of key nutrients such as protein, potassium, and phosphorus, while total phenols and antioxidant capacity remained unchanged, indicating a negative impact of eCO_2_ on spinach’s nutritional quality. However, no experimental studies have yet assessed the effects of CCS leakage on spinach’s physiological morphology, biomass, and nutritional quality. However, a study on lettuce, which belongs to the same leafy vegetable category as spinach, showed that under elevated soil CO_2_ conditions, both morphological indicators and yield decreased significantly; although unit nutrient content increased, the total nutrient content still declined due to reduced biomass, indicating potential adverse effects of CCS leakage on leafy vegetable crops (Huang et al., 2025).

Plant adaptation mechanisms to environmental stress include avoidance and tolerance, which may lead to changes in metabolites and nutritional quality under stress conditions (Badar et al., 2024). The response of spinach to soil CO_2_ leakage stress and its effects on nutritional quality still require further experimental validation. This study hypothesizes that CO_2_ leakage will significantly affect spinach leaf morphology, biomass, and nutritional quality during the CCS process, influencing agricultural production. The main objective of this research is to understand spinach’s response to CCS leakage in terms of (1) morphology and yield and (2) nutritional quality, mainly changes in vitamin C, vitamin E, cellulose, and oxalate content. Although the pot-based approach limits extrapolation to field conditions, this well-controlled study provides the first documented evidence of CO_2_ leakage effects on spinach biomass and key nutritional components.

## 2 Materials and methods

### 2.1 Experiment location and setup

The experiment was conducted at the Shunyi Agricultural Comprehensive Experimental Station, located in Beijing, China (40°5′41.87″N, 116°55′26.76″E). The region has a temperate semi-humid monsoon climate, with an average annual temperature of 11.5°C, an annual sunlight duration of 2750 hours, and an average annual precipitation of 625 mm. The frost-free period lasts approximately 195 days.

**Figure 1:**
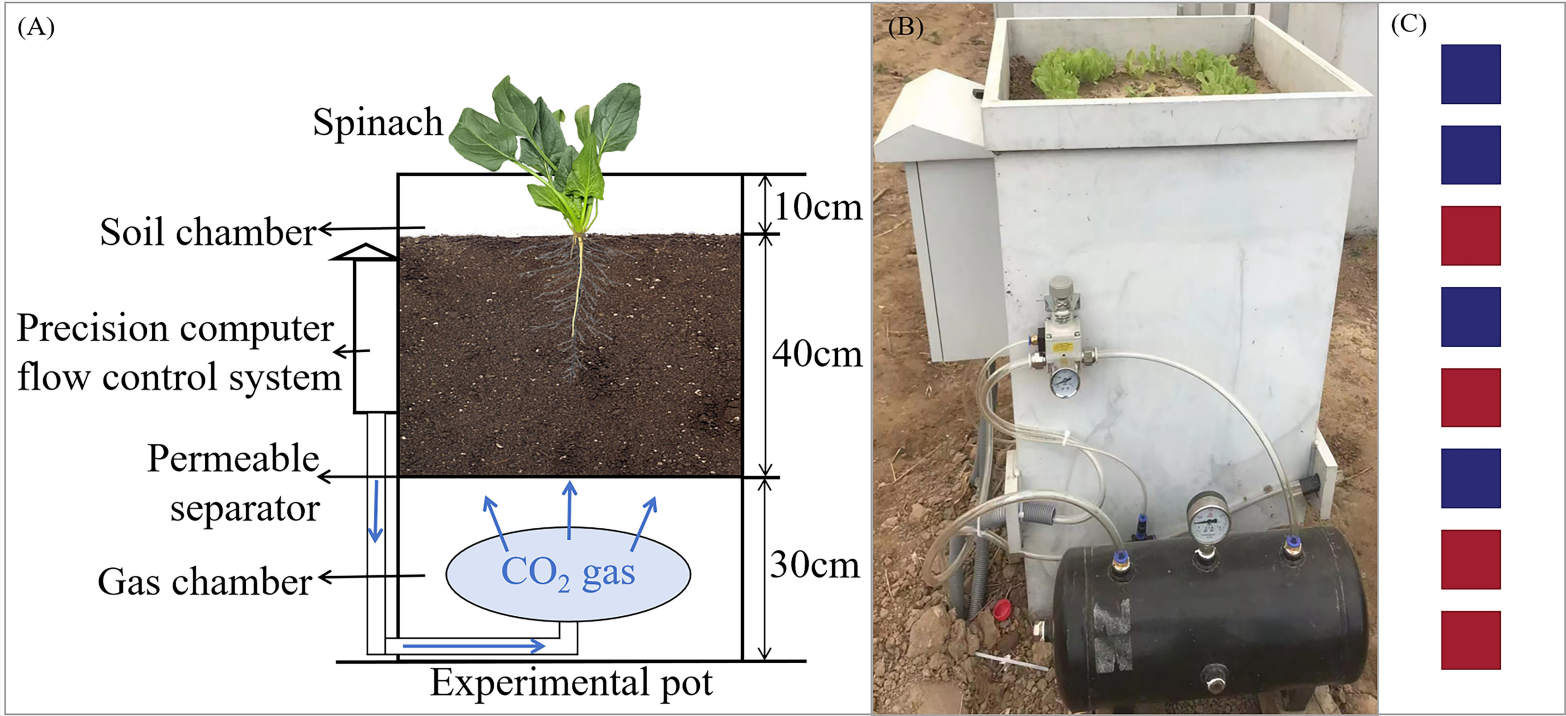
Schematic diagram of the experimental setup and CO_2_ release device. **(A)** Schematic diagram of experimental design, **(B)** the in-site photo of the lettuce leakage experiment, **(C)** pot placement, where red represents G1500, and blue represents CK.

The experimental setup consisted of CO_2_ gas bottles, pots, a CO_2_ flow control system, and gas ducts (**Figures 1A, B**). The dimensions of the pots were 50 cm (length) × 50 cm (width) × 80 cm (height). The internal structure of the pot was divided into three layers from bottom to top: the CO_2_ chamber, the permeable separator, and the soil chamber. The depth of the CO_2_ chamber was 30 cm, while the depth of the soil chamber was 50 cm. CO_2_ from the gas bottles entered the bottom CO_2_ chamber via ducts, and the CO_2_ was then diffused into the soil chamber through the permeable separator. The soil used in the experiment was collected from the surface layer of the nearby farmland and is classified as tidal cinnamon soil with a pH of 7.32. The soil was filled to a depth of 40 cm in the pots. Drainage valves were installed at the bottom of each pot to remove excess irrigation water, ensuring accurate water management during the experiment.

### 2.2 CO_2_ leakage treatment setting and plant management

The experiment, conducted from September 18 to November 8, 2023, included the CO_2_ leakage treatment group (G1500) and the control group (CK). The leakage group had a CO_2_ release rate of 1500 g·m^-2^·d^-1^, while the control group did not receive CO_2_. Each treatment consisted of 4 replicates, totaling 8 pots, arranged in a row from east to west and placed inside an open-top greenhouse covered with a film to prevent external precipitation interference. The distance between the pots was 0.8 m to prevent mutual interference. The layout of the pots for both G1500 and CK was randomized, as shown in **Figure 1C**. Leakage began on October 6, 2023, and lasted until the spinach harvest on November 8, 2023. The CO_2_ injection flux was controlled by a ball valve next to the gas duct and the pot’s precision computer flow control system. The relationship between the flux and rate is as follows:

(1) F=v×ρ/s

Where *F* represents the CO_2_ injection flux (g·m^-2^·d^-1^), *v* is the CO_2_ injection rate (mL/min), *ρ* is the CO_2_ density at atmospheric pressure (approximately 1.977 g/L), and *s* is the cross-sectional area of the pot (0.25 m^2^). When the F was 1500 g·m^-2^·d^-1^, the corresponding injection rate (v) was 131.70 mL/min (Equation 1), with unit conversion applied between days and minutes to ensure consistency of time dimensions. According to our previous study, higher CO_2_ injection fluxes resulted in lower O_2_ concentrations at 40 cm soil depth. Under a CO_2_ release rate of 1500 g·m^-2^·d^-1^, the soil O_2_ concentration was 6.52 vol%, whereas CK exhibited an O_2_ concentration of 17.95 vol% (Zhang et al., 2016).

The spinach variety used was Shubo No. 15, provided by China Vegetable Seed Technology Co., Ltd. (Beijing). Before sowing, all pots were fertilized with nitrogen-phosphorus organic fertilizer as base fertilizer. Spinach seeds were sown on September 18, 2023, and harvested on November 8, 2023. The same management practices were applied to all pots during growth to ensure comparable treatments. **Table 1** summarizes the management practices applied during the experiment.

**Table 1:** Management of the experimental process for potted crops.

Date	Operation	Processing capacity per pot
2023/9/16	Fertilizing	200 g
2023/9/18	Seeding	——
2023/9/19, 21, and 23	Irrigation	1.5 L
2023/9/27	Irrigation	0.75 L
2023/9/30	Irrigation	1.5 L
2023/10/2	Irrigation	3.0 L
2023/10/5	Tinning and Transplantation	——
2023/10/6	CO_2_ injection	132 mL/min
2023/10/9	Irrigation	1.5 L
2023/10/14	Tinning and Sampling	——
2023/10/15 and 19	Irrigation	1.5 L
2023/10/21	Tinning and Sampling	——
2023/10/26	Irrigation	3.0 L
2023/10/31	Sampling	——
2023/11/8	Harvest	——

### 2.3 Measuring the variables

#### 2.3.1 Phenotypic indicators

The phenotypic indicators of spinach included plant height, number of leaves, maximum leaf length, maximum leaf width, and relative chlorophyll value (SPAD). Plant height serves as an indicator of overall developmental status and biomass allocation; the number of leaves correlates directly with photosynthetic capability and yield potential; maximum leaf length and width facilitate the assessment of leaf expansion and allow inference of available photosynthetic surface area; in addition SPAD values offer a rapid, non-invasive proxy for chlorophyll concentration, which further reflects the plant’s nitrogen availability and photosynthetic competence. Integrated monitoring of these parameters enables *in situ* acquisition of multidimensional data on plant physiological status, thereby establishing a robust foundation for evaluating developmental trajectories.

At the start of the CO_2_ leakage treatment, nine spinach plants with similar growth conditions were randomly selected and tagged from each pot to ensure consistent measurements for the same group of plants. Plant height, number of leaves, maximum leaf length, and maximum leaf width were measured twice a week with a ruler, beginning at the onset of CO_2_ leakage. SPAD of the leaves was also measured twice a week starting from October 14, 2023, using the SPAD-502PLUS chlorophyll instrument (Konica Minolta, Tokyo, Japan). For SPAD measurements, one reading was taken from the tip, middle, and base of each selected leaf, and the mean of the three readings was used for analysis. Nine tagged plants were sampled per pot.

#### 2.3.2 Biomass indicators

Three sampling events were conducted throughout the spinach growth period. For each sampling, three spinach plants with similar growth conditions were randomly selected from each pot to measure the leaf area, aboveground biomass, and aboveground water content. The average value of the three plants was used for analysis. At harvest, the same indicators, including belowground biomass, were also measured for the previously tagged plants. The leaf area was measured using the weight method (Shi et al., 2018). Assuming a proportional relationship between leaf weight and leaf area, the total leaf area of each plant was calculated by measuring the weight of leaves with a known area. Aboveground biomass was divided into fresh weight and dry weight. Fresh weight was measured immediately after harvest using a calibrated balance, and dry weight was measured after drying the samples in an oven at 80°C to constant weight (Wu et al., 2022). Aboveground water content was calculated as the difference between fresh and dry weights, expressed as a percentage of fresh weight. Belowground biomass was measured by carefully excavating the spinach roots, cleaning them, and drying the surface moisture with absorbent paper before weighing.

#### 2.3.3 Quality indicators

The quality indicators for spinach included nitrate nitrogen, vitamin C, vitamin E, flavonoids, cellulose, and oxalate content. The concentrations of all quality indicators were measured from fresh samples. After harvest, four samples were taken from each pot, and all indicators except oxalate content were analyzed using the microplate method with a microplate reader (Li, 2000). The nitrate nitrogen content in spinach was measured using a plant nitrate nitrogen reagent kit. In acidic sulfuric conditions, nitrate (NO_3_^-^) reacts with salicylic acid to form nitrosalicylic acid, which appears yellow under alkaline conditions with a peak absorption at 410 nm, and the nitrate nitrogen content was determined by colorimetry (Zhao and Wang, 2017). The vitamin C and vitamin E content were measured using reduction-based assays, where ascorbic acid (AsA) and vitamin E were used to reduce Fe^3+^ to Fe^2+^, forming red complexes with bathophenanthroline (He et al., 2012). Flavonoids were determined using the NaN_2_-Al(NO_3_)_3_-NaOH colorimetric method, where flavonoids form red complexes with aluminum ions in alkaline nitrite solutions, measured at 510 nm (Sultana et al., 2009). Cellulose content was determined using the anthrone reagent method. In this method, glucose derived from fiber undergoes dehydration under concentrated sulfuric acid, forming furfural-like compounds. These compounds react with anthrone reagent to form a blue-green substance, which is analyzed at 620 nm (Updegraff, 1969). The oxalate content was analyzed by chromatographic separation using the Waters ACQUITY UPLC I-CLASS Ultra-performance liquid chromatography and mass spectrometric analysis using the Waters XEVO TQ-S Micro tandem quadrupole mass spectrometer (Gupta et al., 2023).

### 2.4 Data analysis

The primary purpose of data analysis was to explore whether there were significant differences between G1500 and CK in the measured indicators. Data analysis adopted different analytical methods based on the characteristics of the measured indicators.

For phenotypic indicators repeatedly measured during spinach growth, two-way analysis of variance (ANOVA) was used in Python 3.13 to evaluate the effects of Group (G1500 vs CK), Time (measurement dates), and their interaction (Group × Time). When ANOVA indicated significant effects, Welch *t*-test was conducted for pairwise comparisons between G1500 and CK at each time point, and Bonferroni correction was applied to control for multiple testing.

For the other dataset, the Shapiro–Wilk test of normality was performed in Python 3.13. When both CK and G1500 satisfied the normality assumption, Levene’s test was used to assess homogeneity of variances, and based on the result, either an independent sample *t*-test (equal variances assumed) or a Welch *t*-test (equal variances not assumed) was conducted in IBM SPSS Statistics 26. For datasets violating the normality assumption, a permutation test was performed in Python 3.13, as it makes no distributional assumptions and provides robust inference with small or unequal samples.

P denotes the significance level, and P< 0.05 was considered the threshold for statistical significance between CK and G1500. Result figures were drawn using Origin 9.8 software (OriginLab, Northampton, MA, USA).

Additionally, the ANOVA results are summarized in **Appendix Table 1**. The pairwise comparison results of Bonferroni corrections for G1500 and CK across time points are shown in **Appendix Table 2**. While the results of the Shapiro–Wilk test for normality and Levene’s test for homogeneity of variances are presented in **Appendix Table 3**.

## 3 Results

### 3.1 Changes in spinach morphology under leakage

**Figure 2:**
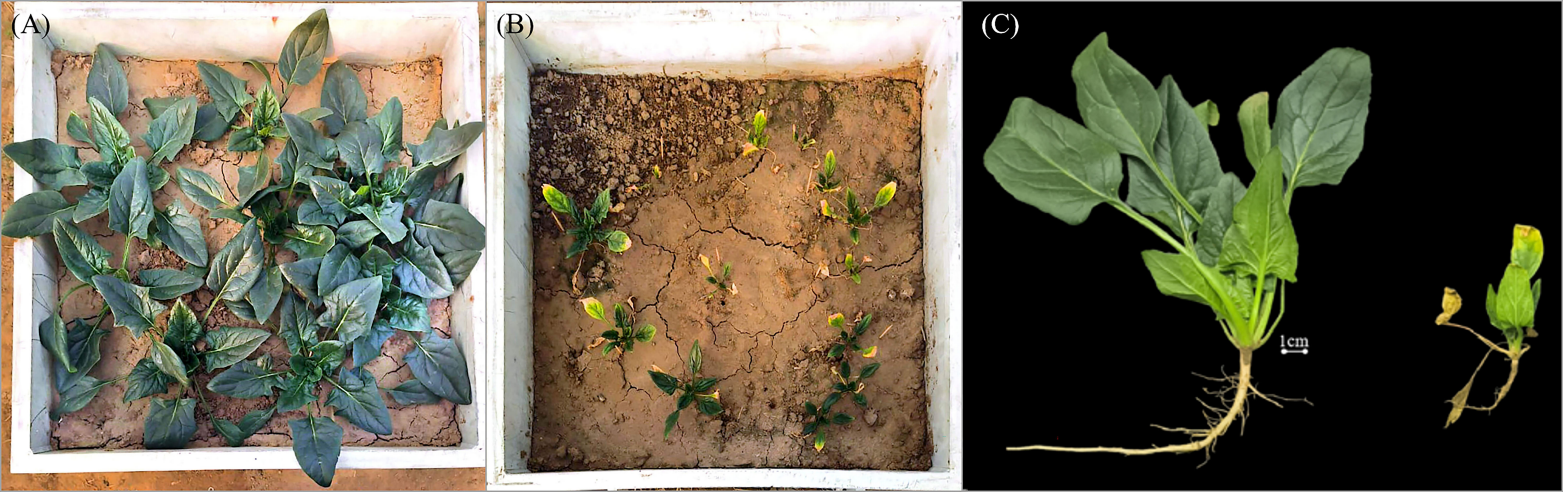
Comparison of the growth of spinach between CK and G1500 during harvest. **(A)** Spinach growth of CK, **(B)** G1500, and **(C)** individual spinach plants, left CK and right G1500.

After 34 days of CO_2_ leakage, spinach was harvested, and significant differences in growth were observed between G1500 and CK. **Figures 2A–C** compare the harvest conditions of the spinach in both groups. In G1500, spinach growth was significantly reduced compared with CK.

**Figure 3:**
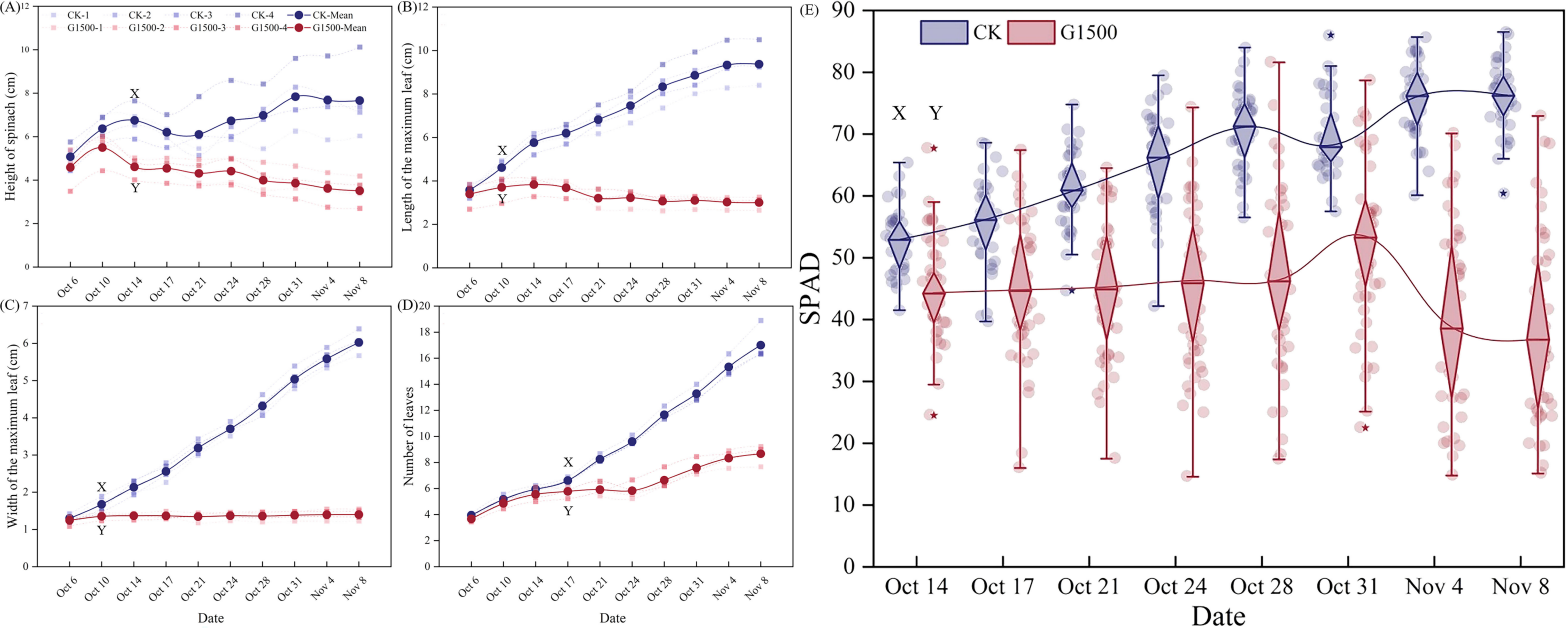
Changes in spinach leaves and SPAD under CO_2_ leakage conditions. **(A)** Plant height, **(B)** maximum leaf length, **(C)** maximum leaf width, **(D)** number of leaves response curve, and **(E)** SPAD box plot. The XY label indicates a significant difference between G1500 and CK from that date, as determined by the independent sample *t*-test. In **(A–D)**, the real point represents the mean of the morphological indicator, and the colored area represents the standard deviation. In **(E)**, “*” represents the deviation value, and the background circle represents the specific numerical value. Blue represents CK, and red represents G1500.

Under the 1500 g·m^-2^·d^-1^ CO_2_ leakage condition, the height of the spinach plants gradually deviated from CK (**Figure 3A**). The spinach of G1500 showed rapid initial growth followed by a fluctuating decline, whereas CK exhibited a continuous and steady increase in height. The height difference between the two groups was substantial, indicating that CO_2_ significantly affected spinach growth. By the end of the experiment, the height of G1500 had decreased by 54.21% compared with CK (N = 36, P< 0.01, **Table 2**). Further analysis using an independent sample *t*-test on the height data indicated a rapid response to CO_2_ leakage. Significant differences were observed as early as the ninth day after the leakage began, indicating that spinach is highly sensitive to elevated soil CO_2_ concentrations, with noticeable changes in plant height.

**Table 2:** Significance analysis of spinach response to CO_2_ leakage at harvest (November 8th).

Observation	Treatment		N	*t*	P	Increase(%)
	CK (Mean ± SD)	G1500 (Mean ± SD)				
Height (cm)	7.66 ± 2.42	3.51 ± 1.15	36	-9.29	0.00**	-54.21
Length of maximum leaf (cm)	9.38 ± 1.74	3.00 ± 0.82	36	-19.85	0.00**	-67.97
Width of maximum leaf (cm)	6.03 ± 1.34	1.40 ± 0.46	36	-19.62	0.00**	-76.76
Number of leaves	17.00 ± 3.01	8.67 ± 2.83	36	——	0.00**	-49.00
SPAD	75.93 ± 5.68	38.59 ± 15.38	36	-13.63	0.00**	-18.38
Leaf area (cm^2^)	226.77 ± 12.55	16.41 ± 3.74	4	-32.31	0.00**	-92.76
Chlorophyll a (mg/g)	0.34 ± 0.15	0.41 ± 0.20	16	1.16	0.26	——
Chlorophyll b (mg/g)	0.33 ± 0.17	0.21 ± 0.10	16	-2.30	0.03*	-35.48
Aboveground fresh weight (g)	11.48 ± 2.29	0.75 ± 0.17	4	-9.35	0.00**	-93.46
Aboveground dry weight (g)	1.30 ± 0.64	0.21 ± 0.08	4	-3.38	0.04*	-84.17
Aboveground water content (%)	89.04 ± 3.69	73.56 ± 5.80	4	-4.50	0.00**	-17.38
Root weight (g)	1.67 ± 0.16	0.07 ± 0.02	4	-19.50	0.00**	-95.83
Nitrate nitrogen (mg/g)	0.25 ± 0.15	0.22± 0.13	16	——	0.64	——
Vitamin C (mg/g)	0.06 ± 0.06	0.18 ± 0.10	16	——	0.00**	185.47
Vitamin E (mg/g)	0.03 ± 0.01	0.06 ± 0.01	16	8.51	0.00**	131.45
Flavonoids (mg/g)	0.54 ± 0.26	3.05 ± 2.20	16	——	0.11	——
Cellulose (mg/g)	2.21 ± 0.91	9.15 ± 4.19	16	6.48	0.00**	315.03
Oxalate (mg/g)	4.94 ± 0.67	2.81 ± 1.32	16	-5.76	0.00**	-43.08

All data were collected at the harvest stage (November 8). Morphological data measured during the experimental period are presented in **Figure 3**. Values are expressed as mean ± SD (standard deviation). N denotes the number of biological replicates. For phenotypic indicators, ANOVA analysis was adopted and Bonferroni correction was applied to adjust for multiple testing across time points. For the other indicators, an independent samples t-test was applied when assumptions of normality and equal variances were met, and a Welch t-test was used when variances were unequal. The *t*-value reflects the standardized mean difference; a larger |*t*| indicates stronger significance. For variables not normally distributed, the permutation test was used, and no t-values are reported (“——”). P indicates the significance level, where * denotes P< 0.05 (significant) and ** denotes P< 0.01 (highly significant). “Increase (%)” shows the relative change of G1500 compared with CK for significant indicators.

The observed indicators include plant height, fresh weight of aboveground biomass, nitrate nitrogen, and vitamin C, among others.

Moreover, CO_2_ leakage inhibited the spinach plants’ maximum leaf length and width. As shown in **Figure 3B**, the maximum leaf length of G1500 grew slowly during the early leakage period and began to fluctuate and decrease from day 12. By harvest time, the maximum leaf length of G1500 had decreased by 67.97% compared with CK (N = 36, P< 0.01, **Table 2**). The decline in leaf length was associated with yellowing and curling of the leaves. **Figure 3C** shows that the maximum leaf width of G1500 displayed a slightly increasing trend, indicating that the high concentration of CO_2_ in the soil had a strong inhibitory effect on leaf expansion. At harvest, the maximum leaf width was significantly reduced by 76.76% compared with CK (N = 36, P< 0.01, **Table 2**). Further analysis showed that significant differences in maximum leaf length and width between G1500 and CK were first observed on the fifth day of leakage. Even more significant differences were noted at harvest, confirming that elevated soil CO_2_ concentrations severely inhibited spinach leaf growth.

**Figure 3D** shows that while the number of leaves in both groups increased overall, G1500 had a 49.00% reduction in leaf number compared with CK (N = 36, P< 0.01, **Table 2**). This suggested that CO_2_ leakage suppressed the leaf formation of spinach. Notably, on day 19, G1500 exhibited a slight reduction in leaf number. As illustrated in **Figure 2C**, several leaves under stress displayed chlorosis and wilting, indicative of senescence.

**Figure 4:**
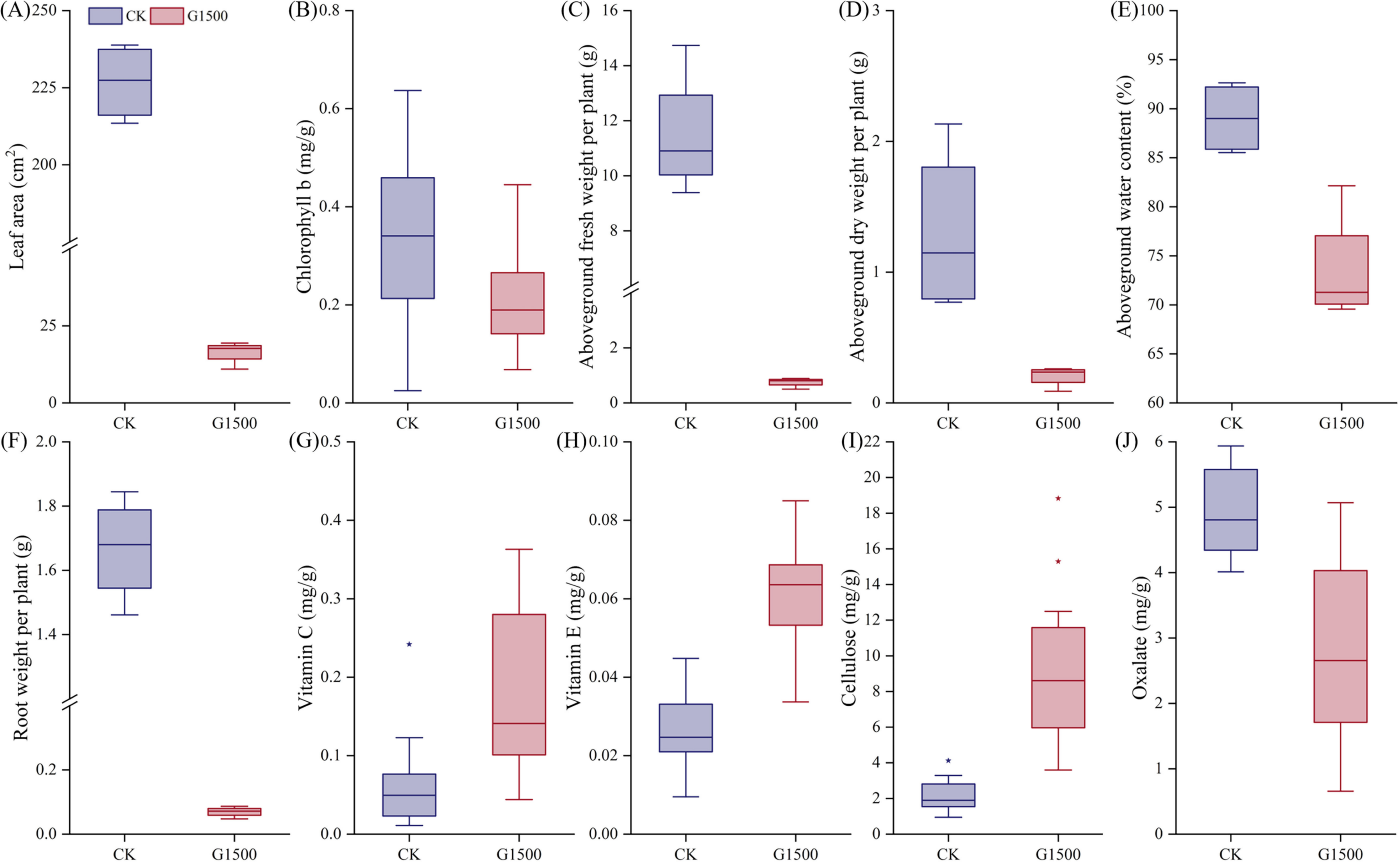
Changes in morphology, biomass, and quality of spinach under leakage treatment during harvest on November 8th. **(A)** Leaf area, **(B)** chlorophyll b, **(C)** aboveground fresh weight per plant, **(D)** aboveground dry weight per plant, **(E)** aboveground water content (%), **(F)** aboveground fresh weight (root weight) per plant, **(G)** vitamin C, **(H)** vitamin E, **(I)** Cellulose, and **(J)** Oxalate box plot. “*” represents the deviation value, and the background circle represents the specific numerical value. Blue represents CK, and red represents G1500.

**Figure 3e** shows the response of spinach SPAD under CO_2_ leakage conditions. During the early stages of leakage, the SPAD of G1500 was lower than CK but did not show significant differences. However, from day 12, after CO_2_ leakage began, significant differences in SPAD were observed, with CK exhibiting a fluctuating decline. At harvest, the SPAD of G1500 was 18.38% lower than that of CK (N = 36, P< 0.01, **Table 2**). Mainly, spinach SPAD under leakage showed a gradual increase followed by a sharp decline, indicating that prolonged exposure to elevated soil CO_2_ concentrations suppressed chlorophyll levels in spinach, leading to leaf yellowing. Further analysis of chlorophyll content at harvest revealed a significant reduction in chlorophyll b content of G1500 (decreased by 35.48%, N = 16, P< 0.05, **Table 2**), suggesting that high CO_2_ concentrations primarily inhibited the synthesis of chlorophyll b in spinach (**Figure 4B**).

### 3.2 Changes in spinach biomass under leakage

At harvest, significant changes in both aboveground and belowground biomass were observed. **Figure 4A** presents the leaf area per plant at harvest. The leaf area of G1500 decreased by 92.76% compared with CK, indicating a highly significant difference and suggesting that CO_2_ leakage severely impaired spinach leaf growth. **Figures 4C, D** show that the aboveground fresh weight of G1500 (0.75 ± 0.17 g) was significantly lower than that of CK (11.48 ± 2.29 g), representing a 93.46% decrease (N = 12, P< 0.01, **Table 2**). The aboveground dry weight of G1500 (0.21 ± 0.08 g) was also significantly reduced by 84.46% compared with CK (1.30 ± 0.64 g) (N = 4, P< 0.05, **Table 2**). Further analysis of the aboveground water content (**Figure 4E**) revealed that CO_2_ leakage significantly affected the water content, which decreased by 17.38% (N = 4, P< 0.01, **Table 2**).

Under CO_2_ leakage conditions, **Figure 4F** shows that the belowground fresh weight (0.07 ± 0.02 g) was much lower than that of CK (1.67 ± 0.16 g), a decrease of 95.83% (N = 4, P< 0.01, **Table 2**). As shown in **Figure 2C**, spinach roots in the high CO_2_ environment underwent morphological changes, with the main root appearing shorter and thinner, and a reduction in the number and length of fine roots. This indicates that CO_2_ leakage restricted the roots’ ability to absorb water and nutrients from the soil.

### 3.3 Changes in spinach quality under leakage

Vegetables contain essential dietary vitamins (Chaudhari et al., 2024), including antioxidants such as vitamins C and E (Stahl and Sies, 1997). In this experiment, CO_2_ leakage significantly increased spinach’s vitamin C and E content (**Figures 4G, H**). Vitamin C increased from 0.06 ± 0.06 mg/g to 0.18 ± 0.10 mg/g, representing a 185.47% increase, and vitamin E increased from 0.03 ± 0.01 mg/g to 0.06 ± 0.01 mg/g, a 131.45% increase (N = 16, P< 0.01, **Table 2**). Vitamin C and E are important components in plant-induced resistance mechanisms, which possess antioxidant properties (Boubakri et al., 2016). The higher increase in vitamin C compared to vitamin E suggested that CO_2_ leakage may more readily induce the production of disease-resistant compounds associated with vitamin C in spinach.

Cellulose, an important component of plant cell walls (Maleki et al., 2016), affects the strength and rigidity of the walls (Taylor, 2008). **Figure 4I** shows a significant increase in cellulose content of G1500, from 2.21 ± 0.95 mg/g to 9.15 ± 4.19 mg/g, a 315.03% increase compared with CK (N = 16, P< 0.01, **Table 2**). This suggested that CO_2_ leakage may stimulate cellulose synthesis, which could help spinach plants cope with the stress induced by high soil CO_2_ concentrations.

Moreover, the oxalate content in spinach decreased significantly under high CO_2_ conditions (**Figure 4J**), from 4.94± 0.670 mg/g to 2.81 ± 1.32 mg/g (N = 16, P< 0.01, **Table 2**), a reduction of 43.08%. Oxalate can accumulate in spinach and interfere with the absorption of calcium and zinc, potentially leading to kidney stone formation (Bsc, 1999). The reduction of oxalate content in spinach under CO_2_ leakage conditions may represent a potential pathway for reducing oxalate levels in vegetables.

According to the results in **Table 3**, a sharp decline in the total content of all measured substances was observed in the G1500 group compared with CK, primarily due to a severe reduction in biomass. The aboveground fresh weight, which serves as the basis for the total content calculation, drastically decreased from 11.48 g in CK to only 0.75 g in G1500. Consequently, although the average concentrations of several key quality indicators (such as vitamin C, vitamin E, flavonoids, and cellulose) increased in G1500, the drastic loss of biomass ultimately led to a substantial reduction in their total accumulation. The total content of nitrate nitrogen plummeted from 2.83 mg to 0.17 mg, vitamin C declined from 0.69 mg to 0.14 mg, and vitamin E content declined from 0.30 mg to 0.05 mg. The total content of oxalate plummeted by over 96.28%, while flavonoids and cellulose were reduced by approximately 63.06% and 72.96%, respectively.

**Table 3:** Comparison of quality indicators between average concentration and total content of spinach affected by CO_2_ leakage.

Observation	Average concentration		Total content (mean)	
	CK (mg/g)	G1500 (mg/g)	CK (g)	G1500 (g)
Aboveground fresh weight	——	——	11.48	0.75
Nitrate nitrogen	0.25	0.22	2.83	0.17
Vitamin C	0.06	0.18	0.69	0.14
Vitamin E	0.03	0.01	0.30	0.05
Flavonoids	0.54	3.05	6.20	2.29
Cellulose	2.21	9.15	25.37	6.86
Oxalate	4.94	2.81	56.76	2.11

## 4 Discussion

Our study investigated the effects of elevated soil CO_2_ concentration on spinach’s growth and nutritional quality. The results indicated that soil CO_2_ leakage significantly inhibited spinach growth, particularly in plant height, leaf morphology, and aboveground and belowground biomass. However, it also promoted the accumulation of specific nutrients, such as a significant increase in the content of vitamins C and E, and cellulose. These findings revealed that the effects of elevated soil CO_2_ on spinach were complex, involving both suppressive impacts and potential activation of plant stress-defense mechanisms.

Nutrient analysis revealed that soil CO_2_ leakage significantly increased the contents of vitamin C and E in spinach by 185.47% and 131.45%, respectively (**Table 2**). This suggests that spinach may activate its stress-defense mechanisms by enhancing the synthesis of antioxidant vitamins under high CO_2_ stress. Both vitamin C and vitamin E play important roles in plant disease resistance, with vitamin C serving as a key inducer of disease resistance. The increase in vitamin C content under CO_2_ leakage conditions aligns with previous studies on plant resistance mechanisms (Boubakri et al., 2016), further confirming the critical role of vitamins in plant responses to stress. Notably, our findings on vitamin C content differ from those of FACE experiments, in which the vitamin C content of vegetables such as carrots decreased under elevated atmospheric CO_2_ (Azam et al., 2013). This discrepancy suggests that soil and atmospheric CO_2_ affected vitamin C synthesis through different mechanisms, potentially due to variations in biomass growth and nutrient absorption patterns.

Additionally, the cellulose content in spinach increased significantly by 315.03% (**Table 2**). Cellulose is a major structural component of the cell wall, and changes in its content often reflect compensatory or integrity responses that impact vegetable quality and stress resistance (Li et al., 2017). The increase in cellulose under CO_2_ leakage likely helped reinforce the cell wall, enhancing the plant’s ability to resist stress. This supported the idea that spinach underwent metabolic adjustments to enhance its tolerance to stress under CO_2_ stress conditions. As Taiz and Zeiger (2015) suggested, plants often adapt to adverse environments by adjusting their cell wall composition, which aligns with the increase in cellulose content observed in this study.

On the other hand, the oxalate content in spinach decreased significantly by 43.08% (**Table 2**). As an antinutrient, oxalate accumulates in spinach and can interfere with human calcium and zinc absorption (Petropoulos et al., 2016). Environmental stressors influence the accumulation of oxalate in plants (Ding et al., 2007; Jiang et al., 2008). Yu et al. (2012) demonstrated that elevated atmospheric CO_2_ levels under heat stress resulted in a 68% reduction in oxalate abundance in cool-season grasses. Similarly, Zhuang et al. (2019) reported that salt stress combined with increased CO_2_ altered metabolite accumulation patterns in the leaves and roots of Kentucky bluegrass, leading to a decrease in oxalate content.

Spinach leaf morphology showed a high sensitivity to elevated soil CO_2_ levels (**Figures 2A, B**), significantly reducing plant height, leaf size, number, and leaf area compared with CK. Notably, plant height rapidly responded, with a significant change observed by the fifth day after CO_2_ leakage. Moreover, the maximum leaf length and width decreased by 67.97% and 76.76%, respectively, while the leaf area decreased by 92.76% (**Table 2**). These results were opposite to those of eCO_2_ experiments, where spinach exposed to high atmospheric CO_2_ for 80 days showed a substantial increase in leaf area (Jain et al., 2007). However, the findings aligned with CCS-simulated soil CO_2_ leakage studies, demonstrating a significant inhibitory effect on plant morphology and biomass (Lakkaraju et al., 2010; Ma et al., 2020b). Our results suggested that elevated soil CO_2_ concentrations had a potent suppressive effect on spinach growth and caused a continuous deterioration in leaf number and health. These morphological changes may be attributed to reduced oxygen availability in high CO_2_ soil environments (Zhang et al., 2016), which affects root respiration and the uptake of water and nutrients.

SPAD showed a significant decline in spinach under CO_2_ leakage conditions (**Figure 3E**). While no noticeable changes were observed early on, SPAD significantly decreased by 18.38% at harvest compared with CK (**Table 2**). This suggested that the inhibitory effect of high CO_2_ on chlorophyll content in spinach intensifies over time, especially under prolonged exposure. This fluctuating decline may be due to disruptions in the chlorophyll synthesis process or accelerated chlorophyll degradation under high CO_2_ conditions (Kumari et al., 2013; Muthusamy et al., 2019). Although the reduced SPAD and chlorosis phenomena suggest a possible decline in photosynthetic performance, no direct measurements of gas exchange, chlorophyll fluorescence, or root respiration were conducted to confirm physiological impairment (Zhang et al., 2020). Therefore, this interpretation remains tentative and should be verified by future studies combining physiological and biochemical assays. Further analysis of chlorophyll a and b content revealed a more significant decline in chlorophyll b (35.48%, **Table 2**), which may lead to a weakened efficiency of the auxiliary light-capturing function of chlorophyll b (Guo et al., 2006). Previous studies on the impact of CCS on leaf photosynthetic capacity support this hypothesis, with findings indicating that high soil CO_2_ concentrations suppress photosynthesis in pasture grass, alfalfa, and soybean (Xue et al., 2021), as evidenced by reductions in photosynthetic parameters such as Pn, Tr, and Gs (Zhang et al., 2015). We expected elevated soil CO_2_ concentrations to weaken spinach photosynthesis, which hindered biomass accumulation.

The results also indicated that soil CO_2_ leakage had a significant negative impact on the aboveground and belowground biomass of spinach (**Figures 4C, D**). Under CO_2_ leakage conditions, the fresh and dry weights of aboveground biomass decreased by 93.46% and 84.46%, respectively (**Table 2**), indicating severe growth inhibition. Furthermore, the aboveground water content decreased by 17.38% (**Table 2**). The high CO_2_ concentration in the soil impaired the growth vigor of the aboveground portion, resulting in a significant decrease in biomass and compromised water regulation of spinach. Below the ground, the root biomass also decreased significantly, with the fresh root weight of G1500 reduced by 95.83% compared with CK (**Table 2**). This result was consistent with findings in alfalfa, where soil CO_2_ leakage at a rate of 1500 g·m^-2^·d^-1^ led to reductions of 43.55% and 66.06% in aboveground and belowground fresh weight, respectively, and a decline in the root-shoot ratio from 1.04 to 0.63 (Zhang et al., 2022). In our study, the reduction in aboveground fresh weight was less than that of the belowground biomass, indicating that CO_2_ leakage had a more pronounced inhibitory effect on the root system.

The experiment also observed significant degradation of spinach root morphology, with the primary roots becoming shorter and thinner and the number, length, and thickness of fine roots decreasing dramatically (**Figure 2C**). The root weight of G1500 decreased significantly by 95.83% compared with CK (**Table 2**). High CO_2_ concentrations severely inhibited root development, restricting the roots’ ability to absorb water and nutrients. This finding is consistent with previous studies, such as Han et al. (2020), who reported reductions of 44.73%, 34.14%, and 19.16% in root length, surface area, and volume of maize, respectively, under CO_2_ leakage conditions of 2000 g·m^-2^·d^-1^. These findings indicate that elevated soil CO_2_ concentrations cause severe physiological damage to spinach roots, hindering their ability to obtain water and nutrients.

Despite the improvements in spinach’s nutritional quality, the significant reduction in aboveground fresh weight (93.46%, **Table 2**) indicated a substantial decline in overall total nutritional content per plant. The reduction in edible yield adversely affected the nutritional and economic value of spinach. The total content of vitamin C and vitamin E decreased drastically by 79.71% and 84.88%, respectively; similarly, a pronounced reduction was observed for cellulose (72.96%) (**Table 3**). These findings highlighted the dual impact of CO_2_ leakage: while it enriched certain nutrients, it significantly impaired spinach growth and marketability. This suggests two non-mutually exclusive mechanistic explanations: a concentration effect due to biomass suppression, and/or active metabolic upregulation under stress conditions. Under atmospheric CO_2_, plants may experience a “dilution effect” (Taub and Wang, 2008), whereby nutrient content per unit biomass decreases as plant size increases (Li et al., 2023; Ahmad et al., 2024). In contrast, soil CO_2_ may inhibit spinach growth, thereby passively increasing nutrient concentrations through a “concentration effect”. However, the potential for stress-induced metabolic activation cannot be ruled out. Previous studies have demonstrated that elevated CO_2_ can active antioxidant and signaling pathways (Yao et al., 2015), upregulate defense-related genes (Loewus, 1999; Wang et al., 2010), and alter carbon allocation and enzyme regulation (Demmig-Adams and Adams, 1996). Moreover, enhanced activity of NADPH- and glutathione-related pathways under CO_2_ stress has been observed in other C_3_ species (Flexas and Medrano, 2002), further supporting possible biochemical adjustments in spinach.

However, this study lacks an in-depth investigation of the dominant mechanisms underlying nutritional changes in spinach under elevated soil CO_2_. While alterations in vitamin C, vitamin E, oxalate, and cellulose contents were observed, the precise metabolic pathways involved remain unverified. Critical evidence regarding key enzymatic activities, intermediate metabolites, and potential pathway regulations is still lacking. Without further analysis of biosynthetic pathways (such as the *L-galactose* pathway for vitamin C and photorespiratory pathway for oxalate), we cannot determine whether the observed changes result from active metabolic regulation or from the passive concentration effect. In addition, similar experiments should be systematically conducted on other crop species to compare metabolic responses and assess whether the patterns observed in spinach are species-specific or represent a broader physiological response.

## 5 Conclusions

This study examined the growth and quality variations of spinach under high soil CO_2_ concentrations resulting from carbon capture and storage (CCS) leakage. Regarding nutritional quality, soil CO_2_ leakage promoted an increase in vitamins C and E as well as cellulose content in spinach leaves, altering the plant’s metabolic pathways. However, the reduction in biomass resulted in a corresponding decrease in the total amount of key nutrients per plant. Additionally, the significant reduction in oxalate content presents a potential avenue for research aimed at mitigating oxalate accumulation in spinach. Elevated soil CO_2_ significantly reduced spinach leaf area, relative chlorophyll content (SPAD), and aboveground fresh weight, severely impairing plant growth and photosynthetic efficiency, thereby affecting overall physiological functions. The reduction in fresh weight of the belowground biomass was more pronounced than that of the aboveground biomass, indicating that the root system was susceptible to high CO_2_ stress.

Our study quantified spinach’s response to soil CO_2_ leakage, showing that while CO_2_ leakage may enhance specific nutritional components, the damage to biomass reduced overall yield and the total amount of key quality components. These findings highlighted the potential threat posed by changes in soil CO_2_ levels to spinach growth and nutritional quality. Our study offers novel insights into how spinach responds to CO_2_ leakage, which can assist CCS project stakeholders in more effectively evaluating the safety of CCS technologies and developing appropriate risk management strategies. Future research could further explore the effects of soil CO_2_ leakage on the growth and quality of other crops to enhance understanding of its broader implications.

## Data Availability

The original contributions presented in the study are included in the article/supplementary material. Further inquiries can be directed to the corresponding author.

## Appendix

**Appendix Table 1** presents the results of two-way analysis of variance (ANOVA) assessing the main effects of Group (G1500 vs CK), Time (measurement dates), and their interaction (Group × Time) on spinach growth parameters, including plant height, maximum leaf length, maximum leaf width, leaf number, and SPAD.

**Appendix Table 1:** ANOVA results for phenotypic indicators during spinach growth.

Observation	Effect	df	sum_sq	F	P
Height (cm)	Group	1	38.44	11.94	0.00
	Time	9	109.77	5.12	0.00
	Group × Time	9	31.63	1.48	0.17
Length of maximum leaf (cm)	Group	1	53.76	20.22	0.00
	Time	9	107.95	5.06	0.00
	Group × Time	9	43.05	3.02	0.00
Width of maximum leaf (cm)	Group	1	50.00	16.03	0.00
	Time	9	193.45	8.88	0.00
	Group × Time	9	65.55	3.01	0.00
Number of leaves	Group	1	5660.08	100.76	0.00
	Time	9	9859.77	24.28	0.00
	Group × Time	9	2731.63	6.74	0.00
SPAD	Group	1	1102.60	15.32	0.00
	Time	7	19239.60	38.18	0.00
	Group × Time	7	14376.73	28.53	0.00

df denotes degrees of freedom, Sum_sq represents the sum of squares, and F is the F-statistic. Larger Sum_sq and F values indicate a more substantial influence of the corresponding factor on the overall variance. Significant P< 0.05 values indicate a statistically significant influence of the corresponding factor.

To control multiple comparison errors, the Welch *t*-test was used to compare means without assuming equal variances, and the Bonferroni correction was applied. The corrected results are presented in **Appendix Table 2**.

**Appendix Table 2:** Bonferroni-adjusted pairwise comparisons (Welch *t*-test) between G1500 and CK across time points.

Date	Height (cm)		Length of maximum leaf (cm)		Width of maximum leaf (cm)		Number of leaves		SPAD	
	*t*	P	*t*	P	*t*	P	*t*	P	*t*	P
Oct 6	-1.54	1.00	-1.13	1.00	-0.78	1.00	-2.47	0.16	——	——
Oct 10	-2.81	0.06	-4.33	0.00**	-3.71	0.00**	-1.58	1.00	——	——
Oct 14	-7.14	0.00**	-8.45	0.00**	-6.56	0.00**	-1.74	0.86	-5.27	0.00**
Oct 17	-5.73	0.00**	-10.17	0.00**	-8.66	0.00**	-3.21	0.02*	-4.60	0.00**
Oct 21	-5.10	0.00**	-12.57	0.00**	-11.79	0.00**	-6.56	0.00**	-7.23	0.00**
Oct 24	-6.03	0.00**	-14.15	0.00**	-12.40	0.00**	-10.36	0.00**	-8.04	0.00**
Oct 28	-8.01	0.00**	-16.17	0.00**	-14.39	0.00**	-11.97	0.00**	-8.52	0.00**
Oct 31	-10.27	0.00**	-17.60	0.00**	-15.85	0.00**	-11.91	0.00**	-6.51	0.00**
Nov 4	-10.85	0.00**	-19.36	0.00**	-18.06	0.00**	-12.85	0.00**	-13.03	0.00**
Nov 8	-9.29	0.00**	-19.85	0.00**	-19.62	0.00**	-12.10	0.00**	-13.33	0.00**

*t* denotes the Welch *t*-statistic, and larger |*t*| reflects greater differences between G1500 and CK. P is the Bonferroni-adjusted P-value, representing the significance level after multiple-testing correction, where * denotes P< 0.05 (significant) and ** denotes P< 0.01 (highly significant). To ensure the validity of the statistical analyses, the assumptions of normality and homogeneity of variances were formally tested for Biomass and quality indicators. The Shapiro–Wilk test was used to assess normality, while Levene’s test was applied to evaluate the equality of variances between CK and G1500. The detailed results of these tests are presented in **Appendix Table 3**.

**Appendix Table 3:** Results of the Shapiro–Wilk test for normality and Levene’s test for homogeneity of variances.

Observation	Date	Treatment	W	P_SW_	P_Lev_
Leaf area (cm^2^)	Oct 14	CK	0.87	0.30	0.01
		G1500	0.81	0.12	
Chlorophyll a (mg/g)	Oct 17	CK	0.90	0.08	0.39
		G1500	0.91	0.10	
Chlorophyll b (mg/g)	Oct 21	CK	0.98	0.94	0.07
		G1500	0.95	0.47	
Aboveground fresh weight (g)	Oct 24	CK	0.89	0.40	0.17
		G1500	0.81	0.12	
Aboveground dry weight (g)	Oct 28	CK	1.00	0.04	0.08
		G1500	0.89	0.36	
Aboveground water content (%)	Oct 31	CK	0.83	0.16	0.08
		G1500	0.78	0.07	
Root weight (g)	Nov 4	CK	0.99	0.74	0.01
		G1500	0.90	0.08	
Nitrate nitrogen (mg/g)	Nov 8	CK	0.87	0.03	——
		G1500	0.76	0.00	
Vitamin C (mg/g)	Oct 14	CK	0.79	0.00	——
		G1500	0.88	0.04	
Vitamin E (mg/g)	Oct 17	CK	0.98	0.98	0.35
		G1500	0.96	0.66	
Flavonoids (mg/g)	Oct 21	CK	0.96	0.59	——
		G1500	0.82	0.01	
Cellulose (mg/g)	Oct 24	CK	0.94	0.29	0.00
		G1500	0.94	0.36	
Oxalate (mg/g)	Oct 28	CK	0.96	0.74	0.01
		G1500	0.90	0.08	

In the Shapiro–Wilk test, W denotes the test statistic and P_SW_ represents the probability of rejecting the null hypothesis of normality; P_SW_< 0.05 indicates a significant deviation from normality. In the Levene’s test, P_Lev_ denotes the probability of rejecting the null hypothesis of equal variances; P_Lev_< 0.05 indicates unequal variances between CK and G1500. When the normality assumption was not satisfied, Levene’s test was not performed.
